# First public dataset to study 2023 Turkish general election

**DOI:** 10.1038/s41598-024-58006-w

**Published:** 2024-04-16

**Authors:** Ali Najafi, Nihat Mugurtay, Yasser Zouzou, Ege Demirci, Serhat Demirkiran, Huseyin Alper Karadeniz, Onur Varol

**Affiliations:** 1 Faculty of Engineering and Natural Sciences, Sabanci University, 34456 Istanbul, Turkey; 2 Center of Excellence in Data Analytics, Sabanci University, 34456 Istanbul, Turkey

**Keywords:** Computer science, Scientific data, Information technology

## Abstract

In the context of Turkiye’s most recent parliamentary and presidential elections (“seçim” in Turkish), social media has played an important role in shaping public debate. It is of utmost importance to capture social media trends during the 2023 Turkish elections, since it uncovers a great deal of information of election propaganda, political debates, smear campaigns, and election manipulation by domestic and international actors. We provide a comprehensive dataset for social media researchers to study Turkish elections, develop tools to prevent online manipulation, and gather novel information to inform the public. We are committed to continually improving the data collection and updating it regularly leading up to the election. Using the #Secim2023 dataset, researchers can examine the social and communication networks between political actors, track current trends, and investigate emerging threats to election integrity. Our dataset and analysis code available through Harvard Dataverse and Github, respectively.

## Introduction

In recent years, political debates are increasingly taking place on social media platforms, and their impact on political behavior has been heavily discussed in the literature^1–4^. This has led to a variety of research in political science, including election forecasting, public opinion, political network detection, and election manipulation^5–14^. As people use social media extensively, various automated approaches are used by politicians, political parties, and voters to garner support and influence a country’s political agenda or manipulating online discourse by spreading fake news and misinformation^3,15–22^. Similarly, citizens’ exposure to social media contributes to the spread of conspiracy theories, which vary by ideological affiliation^23^. In some countries, governments also work with Twitter to censor content and limit the visibility of that content to their citizens^24,25^. In this regard, Twitter is an influential social media platform that can influence citizens’ political engagement, and studying online trends and offline results in elections has become increasingly interesting in recent years^26^.

At a time when Turkiye was preparing for the presidential and parliamentary elections, social media and digital propaganda became increasingly important. The number of Turkish social media users has increased at an unprecedented rate, which can reach a point of addiction among young people^27^. The majority of these young users were the new voters in the 2023 Turkish elections. Using our novel Twitter dataset, we aim to reveal the political trends during pre-election and the campaign periods of the 2023 Turkish elections. Despite the growing literature on social media data and elections, there is still a lack of empirical evidence explaining the key online dynamics during the elections in Turkiye.

In this paper, we present our methodology for collecting a comprehensive social media dataset to study Turkish elections. We operationalize this raw dataset to capture daily user activity, volume of tweets, city-level trending topics, network activity, and ego-centric networks. In addition, we also provide an empirical analysis of bot activity observed on politicians’ social networks.

Turkish politics underwent unprecedented change through referendums, parliamentary/presidential and local elections. Access to alternative sources of information is an essential component of a functioning democracy during the process of free and fair elections^28^. The use of social media has become a significant aspect of public debate on social and political issues, thwarted by the gradual control of mainstream media by government-affiliated corporations^29^. People are finding new ways to connect and gather information through the use of various modalities of online platforms^30^. In Turkiye, this extensive use of social media occurs within the context of debates about popular protests, regime oscillations, polarization, populism, press-party parallelism, and social media manipulation^29,31–39^.

The Turkish elections are of paramount importance to all political parties. Following the adoption of the 2018 “Alliance” article in the electoral law by the Turkish Parliament, two alliances emerged with strong electoral and legislative implications^40,41^. The new election legislation stipulates that presidential and parliamentary elections take place on concurrently for every five years^42,43^. While Parliamentary elections take place using the conventional proportional party-list system, citizens elect the president using a two-round majority method. Moreover, there is a 7 percent threshold for parliamentary elections, which used to be 10% between 1982 and 2022^44^. This leads to a winner-take-all scenario, in case a political alliance win both presidential and parliamentary elections^43,45^. Recently, ruling People’s Alliance does not have power to change constitution unilaterally in the parliament. The Justice and Development Party (AKP, Adalet ve Kalkınma Partisi) and the Nationalist Movement Party (MHP, Milliyetçi Hareket Partisi) would both benefit greatly from winning general elections as this would allow them to remain in power and preserve their alliance^46^. Nationalist Great Unity Party (BBP, Büyük Birlik Partisi) and New Welfare Party (YRP, Yeniden Refah Partisi) are also small members of the alliance.

The main opposition, known as Nation Alliance, consisted of four main political parties including Republican People’s Party (CHP, Cumhuriyet Halk Partisi), Good Party (İYİP, İyi Parti), Felicity Party (SP, Saadet Partisi) and Democrat Party (DP, Demokrat Parti). Two political parties detached from the ruling party AKP, Democracy and Progress Party (DEVA, Demokrasi ve Atılım Partisi) and Future Party (GP, Gelecek Partisi) also support Nation Alliance. The third group is represented by People’s Democratic Party (HDP, Halkların Demokratik Partisi), which is composed of mostly Kurdish electorate and various leftist political parties and they form another alliance along with The Workers’ Party (TIP, Türkiye İşçi Partisi) and 5 other parties, called Labour and Freedom Alliance^47^.

The Turkish parliamentary and presidential elections occured at a time when economic and political problems and polarization are at their peak^48^. It was during this period that all actors are trying to influence public opinion via social media, either by attracting citizens or provoking negative campaigns against their opponents. In general, electoral campaigns intersects electorate’s social media exposure, by which ideological and political groups buy the propaganda, information and conspiracy.

This electoral process is supported by the extensive use of social media by the population. According to internet usage statistics published annually by DataReportal, Turkiye has had an increasing number of internet users and social media penetration since 2019^49^. According to their analysis Turkiye had 59 million internet users and over 9 million Twitter users in 2019. These numbers increase to 71.3 million and 18.5 million, respectively. In this paper, we examine the 2023 Turkish general elections by highlighting the key social media trends for the pre-election and campaigning processes. We adopt Norris et al.’s^50^ phases of election, which include the pre-election, election campaign, election day, and post-election periods. Each period features different modes of political dynamics. For example, the first two phases include negative campaign strategies, election promises and individual criticism towards candidates, debates over election laws, media portrayals of each party, and campaign financing. Therefore, the timing of our study is also suitable for describing the online political behavior of citizens and politicians, i.e., the dynamics of citizens’ political behavior in social media can be best captured during this phase.

This work presents a novel data scrutinizing specifically Turkish General Elections in 2023 and it is the first and the most comprehensive dataset for 2023 elections. One of our goal is to share this study to make comparative studies possible with elections in other countries. Projects like DigiWorld (https://digidemo.ifkw.lmu.de/digiworld/) and Comparative Study of Electoral Systems (CSES) (https://cses.org/) are examples of those. There are also efforts to build data collection pipelines to collect multi-platform social media data to track election campaigns^51–53^. In addition to data collected from social media, researchers also gather datasets for studying electoral campaigns^54^ and voter fraud claims^14^. Comparative analysis are important since they are also useful to determine possible empirical schemes to compare political entities’ campaign tactics^55^ or how parties or politicians can mobilize their voters^56^.

Previously, several researchers have looked at how to use Twitter data for political analysis^29,57–62^. The techniques used in these studies include a wide range of computational tools, including network, sentiment, and content analysis. Our contribution to the literature is based on three key pillars of big-data analysis. As a first step, we provide a novel dataset on Twitter and other online sources that is essential for understanding the main dynamics of the 2023 Turkish parliamentary and presidential elections. Using Twitter Developer APIs, our dataset captures both user data and influential political figures in Turkiye. Second, we provide an initial analysis of social networks, daily changes in political figures’ followers, trending topics, the number of daily tweets from individual users, and party membership data by time. Thus, our raw data combine empirical and technical aspects of computational tools with political science. Finally, we discuss the implications of our study for further operationalization of the dataset. We contribute to the literature not only by providing a structured dataset, but also by setting a research agenda for how it can be used to describe existing trends before and during an election.

## Methods

**Figure 1:**
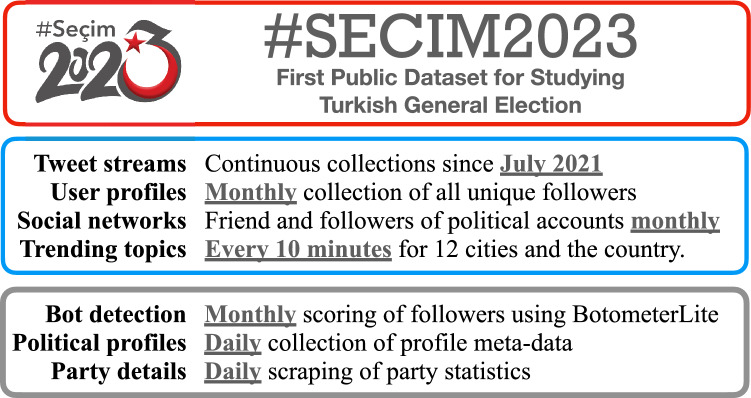
Summary of #Secim2023 dataset. Our dataset captures content produced about politics and election through online streams. It is also updated frequently to track changes in underlying social network and trends.

In analyzing conversations about elections and political debates, we rely on predetermined keywords and users. Using available resources, we aim to capture a holistic picture of Turkish elections on Twitter. Data collection in this project uses Twitter API versions v1.1 and v2.0. Our team has access to the standard developer API and elevated access via the Academic API. We use the tweepy and twarc libraries for Python to systematically access the Twitter API. We also developed custom web scrapers to download additional information such as party membership statistics. Schematic in Fig.  1 summarizes the different data sources.

*Trending topics* Twitter provides “trending topics” on the platform to share important conversations. These trending topics can be hashtags or phrases, and are available at the city and country level as well as globally. We use Twitter’s Trending Topics API to collect hashtags and phrases for 12 cities available in Turkiye and country-level trends. We collect trends every 10 min to systematically track changes in conversations. Twitter API requires WOEID (Where On Earth IDentifier) to collect trending topics for certain geographical region using their trends/place endpoint. For instance the world has WOEID equal 1 and Turkiye has 23,424,969. Twitter provides trending topics for limited number of countries and their cities, so the API provides list of available cities on trends/available endpoint.

*Twitter annotation streams* Twitter Academic API introduced tweet annotations where named entities comprised of people, places, products, and organizations are automatically detected^63^. Twitter use these entities to link with various topics including politics. We selected those context annotations about politics (context:35.*, context:38.*, context:88.*) and filtered the ones that are written in Turkish. Since the volume of activity is quite significant and the Academic API limits us with 10 million tweets per month, we collect random sample of 25% of the retweeted content and keep all original tweets, quotes, and replies.

*Streaming API* Although the entity annotation feature is useful for data collection on a particular topic, our initial analysis shows that Twitter’s entity detection system systematically biased towards the members of the current government. Since we want to capture all political discussions in our dataset, we create our own keywords and users lists for collecting data from the streaming API. Our collection of political users includes party leaders, mayors of the major cities, and members of the Grand National Assembly from the last two terms. Although this list is comprehensive and currently covers 936 different users, we regularly update our list to capture new actors, organizations, and media channels and the latest list reached up to 1589 accounts. This stream remained active until Twitter retired the V1 filter endpoint. From March 17th to June 22nd (the date Twitter Academic APIs are discontinued), we defined stream rules to work with Twitter API V2 and replaces annotation stream with these new data collection to manage ratelimits more efficiently. The most recently incorporated API V2 captures all direct tweets about candidates and party leaders, but samples retweeted content with 10% sampling rate.

*Social networks and profiles* The Twitter API can provide social network connections. We have collected both friends and followers of the political accounts. The API provides these connected accounts in chronological order, based on when the edge was created. Since Twitter API limits for social network quite limited, each request returns 5000 users and popular accounts require thousands of API calls to capture the entire network. Since the rate limits allows 15 requests within a 15 min windows, this is an expensive data collection task. Currently, we try to recollect networks monthly. Once we have network representations, we collect profile objects of these users for further analysis. In this dataset, we provide meta-data about account profiles such as id, name, screen name, account creation time and statistics such friend, follower, and tweet counts at the time of data collection. We recollect all profiles monthly, to capture changes in the profiles, identify deleted or suspended accounts.

*Party membership* Statistics about party members are shared on the website for “General Prosecutor Office of the Supreme Court of Appeal” from the website https://www.yargitaycb.gov.tr/kategori/117/siyasi-partiler. We developed a scraper for this website to collect statistics about memberships daily; however, the website itself updated these statistics less frequently and with non-regular intervals.

## Technical validation

**Figure 2:**
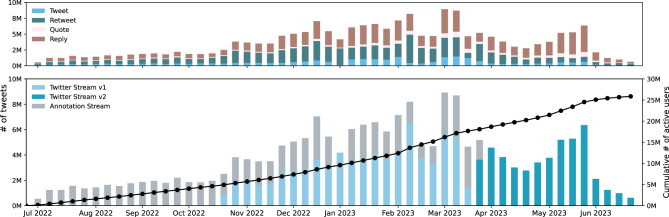
Temporal statistics of streaming datasets. We track number of weekly tweets for different streaming APIs and the unique users sending those tweets.

Our dataset is comprehensive and unique to Turkish elections in that it collects trending topics, tracks political accounts, and extracts networks and additional signals from these entities. Our data streams provide Turkish tweets that contain political entities or relevant keywords. On average, we collect about 300,000 tweets per day posted by more than 25 million accounts in the last 12 months, as shown in Fig. 2. In terms of the type of content, retweets and replies are the predominant interaction types, as they serve to disseminate arguments and debate issues, respectively. Overall, 54% of the content are retweets and 14.5% are replies to posted tweets. In our dataset, we also provided meta-data on association between different tweets, their users, and creation times to make network analysis easier using our dataset.

**Figure 3:**
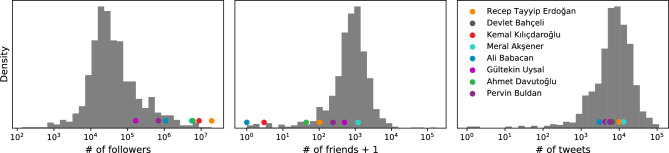
Profile statistics of political actors. Comparing profile metrics such as number of friends, followers, and posts for accounts in our collection.

We also track the profile statistics of political accounts daily. We can compare their profile characteristics as shown in Fig. 3. Exemplar politicians we selected produce average level of content, but are more popular than the rest of the political figures and they are more selective for their friends.

**Figure 4:**
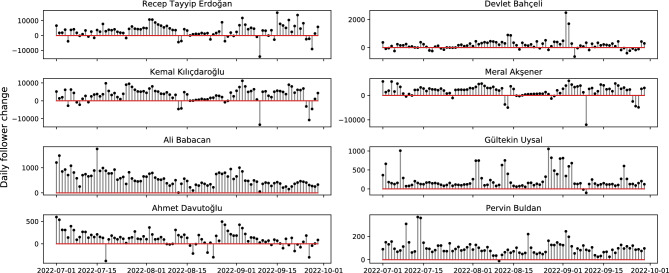
Daily follower changes of political figures. We collect profile information of politicians daily and we can monitor changes in their profile statistics daily.

Since we track the daily changes of politicians profiles, we can look at the daily changes of their followers as a time series. In Fig. 4, we can point the correlated changes of follower counts. For instance politicians from different political ideologies such as Recep Tayyip Erdogan, Kemal Kilicdaroglu, and Merak Aksener lost nearly 10,000 followers on September 9th, 2022. This significant change can be due to deleted accounts or result of an automated coordinated activity. By analyzing these temporal patterns and the profiles of these unique followers, we can address questions about their role for these political figures.

### Trending topics in Turkiye

Trending topics frequently reflect important events such as sporting events, political debates, or TV in Turkiye. They are also shown to be manipulated by means of automation, and a recent study suggests that 47% of the top 5 daily trends are generated by astroturfing attacks^64^. Considering this significant vulnerability analysis of trending topics will be more important for the elections.

**Figure 5:**
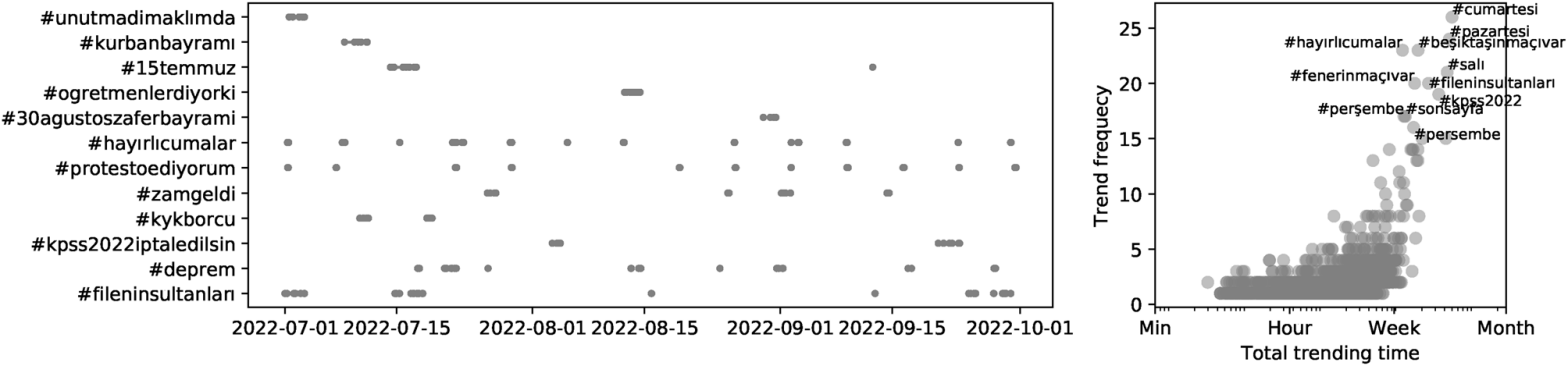
Temporal characteristics of trend topics. Trending topics of a particular location (Turkiye in this example) can be visualized as a timeline (left). Frequency and trending time can also be calculated for all trends to identify outliers (right).

To track manipulated trending topics and capture important events, we collect those trends regularly. In Fig. 5(left), we show sample of trending hashtags that appeared in the top-10 list for a significant period. We analyzed the trends in Turkiye and studied their frequency and durations (see Fig. 5(right)). Some of these hashtags point important days such as #unutmadimaklimda to commemorate Sivas massacre happened in July, 2nd 1993 or #30agustoszaferbayrami for celebrating Victory day. There are also more generic hashtags that represent days of the week or football games repeat in regular intervals, while most hashtags stay in the trending list for less than a day. It is important to investigate emerging hashtags that reach to significant level of visibility and amplification. For instance, unfortunate events of Feb 6th Earthquakes in Southeast of Turkiye immediately observed in the trend dataset. Relevant hashtag #deprem (earthquake for Turkish) was trending countrywide for more than 2 days. Although our trending topic data records only the entity names, Twitter API can be used to collect tweets and unique accounts spreading these hashtags or phrases.

**Figure 6:**
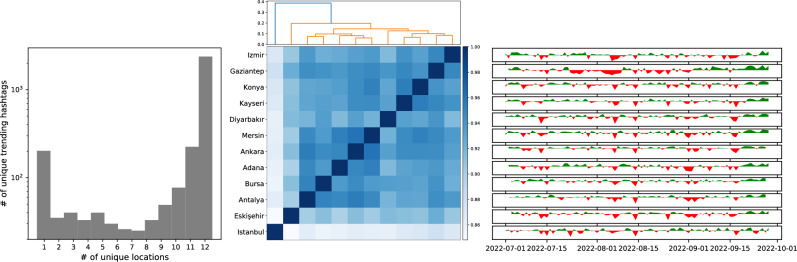
Trend topic similarity at the city level. Some trends are localized meaning appeared only few cities and other reach country-wide popularity (left). Similarities of the trending topics between cities (middle) and their similarity with the Turkiye trends (right).

Since Twitter provides trending topics at the city level and countywide, we can examine the relationship between them. Previous research suggests that there are two mechanisms that drive trend propagation: local diffusion processes and global transmission of trends due to travel hubs^65^. We observed that some trends remained localized; however, the majority of trends achieved nationwide popularity, indicating that the themes spread quite efficiently among the population as shown in Fig. 6(left). We can investigate which cities set these trends or adopt others by studying the emerges of the hashtags and phrases on different geographical locations. Some cities, such as Istanbul, Eskisehir, and Diyarbakir, stand out in terms of their unique trends, while others tend to cluster based on geographic and cultural similarities (see Fig. 6(middle)). Another explanation could be the “newsworthiness” of various cities in Turkiye. Emre Kizilkaya investigated how different regions are covered in the news^66^. He found that the inner regions of the Aegean Sea and the Anatolian provinces (with the exception of Ankara), as well as the Black Sea and southeastern regions of Turkey, are less represented in the news. People living in these cities may also access trending topics from other cities and share their location, making them trend adopters. We can also track over time how similar these local trends are compared to national trends in Turkiye (Fig. 6(right)).

### Social and information network of Turkish politicians

Network analysis allows us to observe organizations at the macro and meso levels. These networks can represent a static view of the organization, but can also capture changes over time by using data from different time intervals. Our dataset provides information to create and analyze at least three different networks.

*Social network* In this representations nodes correspond to social media users and edges capture their relationships. These relationship can measure their shared followers, content similarity, etc. We regularly collect friends and followers of political accounts, we can build an egocentric network of politicians as well as a similarity network of these political accounts based on their common followers.

*Information diffusion* This network captures how information spreads through users by tracking interaction types such as replies, retweets, quotes, and mentions. Nodes can be different tweets or users, and edge weights can represent time delays or frequency of interactions. An aggregated diffusion network represents users as nodes and interaction frequency as edge weights. Alternatively each tweet can be a node and edges contains details about time delays or means of interactions.

*Hashtag co-occurrence* Based on the co-occurrence of different memes such as hashtags, URLs, and phrases, we can create a network representation of these memes to show community structures. This is a common approach to study underlying contexts and their association with each other^67,68^. Community detection approaches are frequently applied to these networks to study themes discussion online.

**Figure 7:**
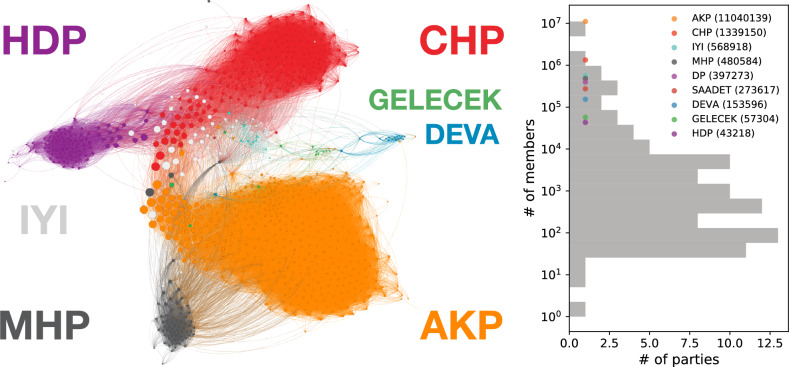
Party networks and memberships. Network nodes present politicians colored to represent their party memberships (left). Registered voter statistics for all Turkish political parties and voter numbers for exemplar parties presented (right).

In Fig.  7 we represent a network of political accounts. In this network, the nodes represent different political figures coloured by their party affiliation. We computed the edge weights as Jaccard similarity between their followers to represent the similarity of their audiences. We applied an additional filtering step to remove edges with low weights by applying a threshold, and used the ForceAtlas2 algorithm as a layout to locate nodes in two-dimensional space. In this network, politicians belonging to the same parties tend to cluster together as partisans share multiple politicians from the same parties. The community organization of this network also provides insight into Turkish politics, as AKP (orange) and MHP (dark grey) represent the People’s Alliance, and CHP (red), IYI (light grey) from the Nation Alliance cluster together. DEVA is also close to Nation Alliance regarding follower trends. The HDP (purple) has distinctly separated from the other two groups, while its politicians share more followers with the politicians from CHP. Later in the elections, the HDP decided to support the People’s Alliance candidate and did not run its own candidate. We can also note that the parties DEVA and Memleket, which were between the two alliances in the figure, left the People’s Alliance after the election. The network representation also captures left and right polarization as well as the ethnic separation.

**Figure 8:**
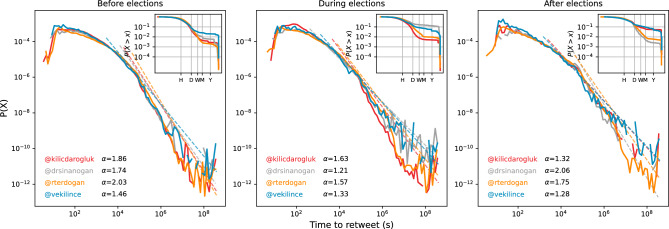
Retweet lag for politicians’ content in different periods. Time between politicians’ tweets and their retweets shown for three different period of the election. Cumulative distributions presented in the inset figures.

Social media users can also engage with politicians’ content. Their followers can retweet these messages to spread them among their followers, or reply to tweets to make their points. Time lags between politicians’ tweets and retweets can point engagement rates of their audience. In Fig. 8, we show the distribution of retweet lags for three different time periods for the election. We also estimate the exponent for powerlaw distributions to compare politicians among themselves and across different time periods. In this analysis, we can first compare the scaling exponents for the period before the election and between two elections. The exponents are smaller during the election period compared the campaigning period for all candidates, suggesting that users are engaging with older content, while at other times they are more responsive to current content as pointed out by larger exponents. When we analyze the cumulative distributions from the inset figures, we find that Sinan Ogan and Muharrem Ince have about 10% of their retweets on their older content. This is particularly pronounced for Sinan Ogan, as he was opposed to Recep Tayyip Erdogan but later supported him in the second round of the election. Users find his older tweets to point out the hypocrisy. After the election, victorious leaders have a greater scaling exponents as their supporters also focus on celebrating their current content and respond more enthusiastically.

**Figure 9:**
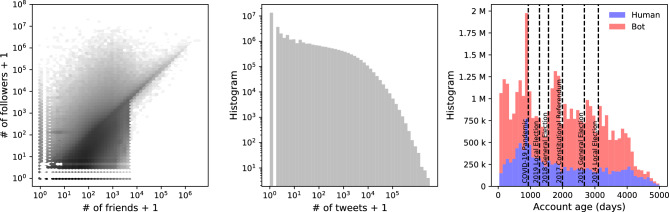
Analysis of followers. Friend and follower statistics (**a**) and content production measured by tweets and retweets (**b**) for the followers of politicians presented. We also compared their account creation times and and their use of automation (**c**).

Since social networks consist of regular users, their participation in political discourse or engagement with politicians is also important for understanding their representativeness to voters. In Fig. 9, we present basic statistics about the following accounts. Most of these accounts have fewer than 1,000 friends and followers. Their productivity follows a heavy-tailed distribution, as most accounts have less than 10 tweets, while very few have more than 100,000 tweets. This discrepancy in content production suggests automated activity and suspiciously almost 10 millions of these accounts have no tweets and their only activity is following other accounts^69–71^. Since our dataset captures these followers at regular intervals, we can also examine the deleted network nodes over time.

Analyzing users’ following political accounts, we observe when they were created in Fig. 9c. We find that more bot accounts than human accounts are created every day since the beginning of 2010. We observe an increase in the creation of automated accounts prior to the 2014 local elections and the 2015 and 2018 general elections. We also note that during the pandemic, the number of human and bot followers increased, and some of these accounts may promote anti-vaccine sentiment and could be repurposed to support certain political ideologies in the 2023 Turkish election. Considering our earlier observations of fluctuations in the number of followers and anomalous accounts with extreme content production, it is reasonable to suspect the existence of social bots.

### Automated activities in political networks

There are several online tools for detecting automated activity, and this is an active area of research given the increasing involvement of automation in political discourse^18,69^. In this work, we use the Botometer system and its light-weight version called *BotometerLite* to evaluate Twitter accounts^72–74^. This system analyzes user profile information to assess the bot likelihood of an account. Although BotometerLite uses a more restricted list of features, it can scale up to evaluate millions of accounts and only requires the profile information which we already collect for the #Secim2023 dataset.

**Figure 10:**
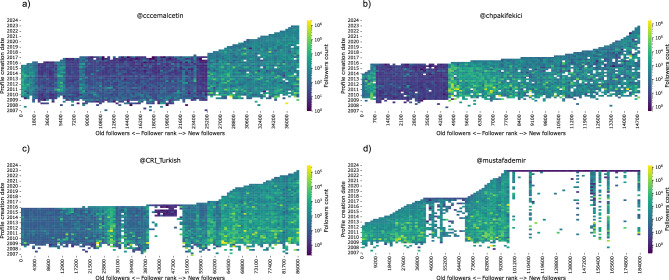
Anomalous followers. Follower counts for each user groups based on their account creation times (y-axis) and follow rank (x-axis) analyzed for different accounts. We present exemplar accounts that exhibit patterns for inactive followers (**a**,**b**) and rapid changes of followers with self-similar account groups (**c**,**d**).

Using the followers of a politician we can study the patterns of anomalous followers. In a recent paper, we study journalist accounts and their followers^75^ and recently developed and unsupervised learning approach to automatically detect anomalous followers^76^. In that study, we encountered anomalous follower patterns, suggesting that fake followers might be purchased to either increase an account’s popularity or to manipulate their journalistic activities. We apply this method to our dataset and investigate suspicious follower patterns in a follower network of politicians. For this analysis, we use the list of followers provided in chronological order of followers and their profile metadata. In Fig. 10, we share four exemplar accounts that exhibit different patterns of anomalous followers. Accounts are ranked along the horizontal axis based on their follow times and vertical axis presents account creation date of these followers. Politicians in Fig. 10c and d have followers that are created in a very narrow but recent time interval and follow the politician around the same time. Other examples show dormant followers that have very few followers.

Since social bots can be used to promote politicians, parties, and their agendas, their impact can be positive for their campaigns^20,21^. However, there are also alternative scenarios in which social bots are used to target politicians, manipulate their engagement rates, and paint a misleading picture of their online presence^75,77^. In this sense, we do not claim that politicians buy social bots for their own benefit. There may be multiple sources of generating social bots, including international actors that can carry out different manipulation techniques to mislead. It is important to consider alternative explanations and collect more evidence to support each claim. Here, we present a brief analysis conducted for 4 major political parties and their politicians.

**Figure 11:**
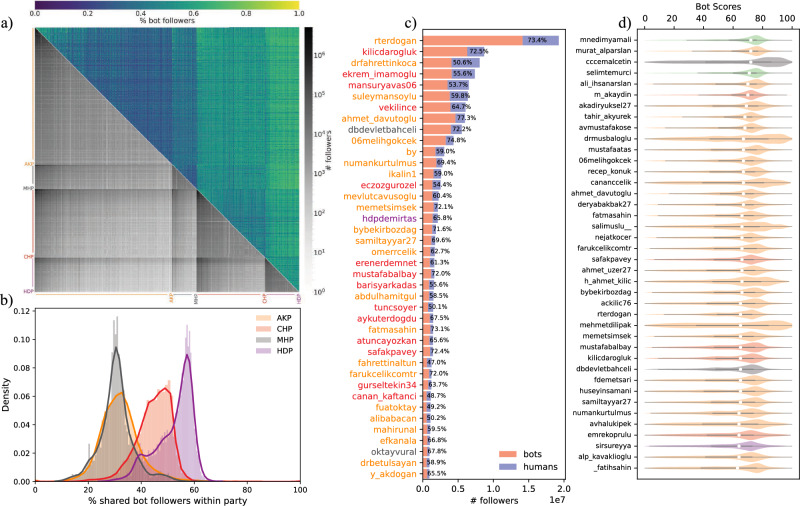
Social bot analysis. Politicians from different parties compared based on number of shared followers and percentage of bots shared (**a**). Percentage of shared bot followers are also analyzed within parties (**b**). Individual bot follower statistics investigated by ranking accounts with most followers (**c**) and highest percentage of bot followers (**d**).

When social bots are studied in the context of politics, the initial question is usually who has the most bot followers. In Fig. 11, we analyzed individual politicians and their aggregate statistics for their parties. Since the prevalence of bot followers can be measured in both exact numbers and percentages, we presented both quantities. In Fig. 11 a, we grouped politicians by their parties and calculated shared number of followers in the lower triangle of the heatmap and plotted the percentage of bot followers among them in the upper triangle of the heatmap. We can see that politicians have more shared followers within their party; however, politicians from CHP and HDP have stronger connections amoung their fellow party members. The percentage of social bots also follows a similar pattern; social connections with CHP and HDP contain more bot accounts. This observation of party connections becomes clearer in Fig. 11b. It is important to remember that these bots can work for or against these politicians, and this is a research question that we are currently investigating.

When we inspect the most popular individuals (see Fig. 11c) and the ones with the highest percentage of bot followers (see Fig. 11d), we observe different sets of names. Popular accounts known to be targeted by social bots to influence their online activities or amplify their engagement metrics and popularity^75^. We observe a similar result; the popular accounts usually have more than 50% bot followers. These accounts with high number of followers are mainly from AKP and CHP parties, with only 3 exceptions in the top-40 list. Alternatively, we can also rank politicians by the percentage of bots among their followers. In this figure, we show the distribution of bot scores and the median bot score for these accounts.

An important research work deals with the role of these bot followers. We will investigate information dissemination network using natural language processing tools to determine whether the observed bots work for or against these politicians over the course of the election. The different perspectives we provide in this dataset may raise additional research questions about the behavior of political figures, how they gain online support, and the campaign practices of political parties. We also see the potential of our dataset to study online manipulation and the use of automation to influence social media users. We hope that researchers will use this data and even combine it with their own tools and datasets to address novel research questions.

## Usage notes

In this paper, we introduce the #Secim2023 dataset and provide a preliminary analysis to highlight potential research questions that can be addressed with this dataset. For further empirical purposes, researchers can use our data for a variety of research purposes, including network analysis, machine learning applications to predict public sentiment and topics, user demographic data, and election results. During COVID-19 pandemic, scientific community collect and share valuable datasets with the research community to expedite international research efforts^78–80^. Recent changes in the social media platforms and their user bases make cross-platforms comparisons more valuable. There are recent projects to gather multi-platform datasets for comparative analytics^52,53^.

Influence of automated accounts require more in-depth analysis where content analysis, sentiment towards certain parties should be studied with a political science perspective. Our dataset can create opportunities for such interdisciplinary research. The dynamic nature of this dataset will support researchers to capture different aspects of the election. Using this dataset, researchers can answer several important research questions about election campaigns, manipulation activities, online trends, etc. Our team will be using this dataset to work on primarily the following tasks: (1) tracking evolution of topics and the rate of content production various topics, (2) analyzing followers of prominent politicians and study their social bot followers and report changes in their audience, and (3) developing tools for early-detection of online manipulation, predicting party affiliations and user demographics.

We believe that the #Secim2023 dataset will be a valuable resource for researchers developing natural language processing systems for Turkish and investigating behavior of Turkish speaking social media users using machine learning^29,58,60,81–83^. We have collected #Secim2023 dataset and created the metadata that can be made available to researchers. We have obtained approval from the Sabanci University Ethics Committee (*#FENS-2022-19*) to conduct this research and all experiments were performed in accordance with relevant guidelines and regulations. We also follow the guidelines of the Twitter Developer Agreement for sharing data collected through their APIs. Upon reasonable request, we can also share more detailed datasets for research projects. We hope other researchers complement their approaches with online data. There are vast opportunities for interdisciplinary research using computational social science tools between computational and social science researchers.

*Limitations* Although we present the most comprehensive and unique dataset to study the 2023 Turkish elections, the dataset may have some limitations. First, our Twitter stream tracks activity across a manually curated list of accounts. We have attempted to capture political figures that represent all of Turkish politics. These collection streams may have biases due to Twitter^84,85^. Second, the Twitter developer agreement limits our ability to share raw data, but researchers can use the Twitter API to rehydrate the original data as long as the tweets are not deleted in the meantime. Finally, Elon Musk’s acquisition of Twitter created some changes in the platform, the availability of data, and the participation of automated accounts in political discussions^86^. Since the acquisition, Twitter retired some of its API V1 endpoints, so we use Twitter’s Academic API to collect streaming data instead of filter endpoints. Fortunately, our API access remained active until a month after the elections. Lastly, researcher can use this dataset and enrich their analysis by using third-party services like m3inference^87^ for demographic inferences and Botometer^74,88^ or other systems for identifying inorganic accounts. These system may require specific format of data and researchers may need to rehydrate data from Twitter. However, the data collection methodology and analysis can be applied to other election campaigns.

## Data Availability

We provide Github code for the analysis on this paper: github.com/ViralLab/Secim2023_Dataset. The dataset is also publicly available on Harvard Dataverse^89^ to follow guidelines of FAIR principles. For privacy reasons, we can only share a limited amount of data; however upon reasonable request through email to corresponding author, we can share detailed raw data for research purposes. Twitter’s Developer Agreement & Policy limits us sharing the full dataset collected through their API. This limits our ability to share entire information about tweets, but instead we provided tweet IDs that can be rehydrated using Twitter’s API. Software packages like Hydrator^90^ or Twarc^91^ can be used to systematically download data. Unfortunately, deleted tweets will not be available when collected from API.
